# Sturge-Weber Syndrome: A Case Report

**DOI:** 10.31729/jnma.8344

**Published:** 2023-11-30

**Authors:** Sunil Timilsina, Bishal Kunwor, Suchit Thapa Chhetri, Sanath Nepal, Khusbu Sedhai

**Affiliations:** 1 Department of General Practice and Emergency Medicine, Shree Birendra Hospital, Chhauni, Kathmandu, Nepal; 2 Nepalese Army Institute of Health Sciences, Sanobharyang, Kathmandu, Nepal; 3 Department of Internal Medicine, Manmohan Cardiothoracic Center, Maharajgunj, Kathmandu, Nepal

**Keywords:** *brain*, *case reports*, *port-wine stain*, *seizures*, *Sturge-Weber syndrome*

## Abstract

Sturge-Weber syndrome is a rare congenital neurocutaneous syndrome with an incidence of 1 in 50000 characterised by facial capillary malformation and vascular anomalies in the brain and eye. We present the case of a five-year-old child diagnosed with Sturge-Weber syndrome. The patient exhibited high-grade fever, headaches, and generalized tonic-clonic seizures. The history revealed a port-wine stain on the face and a history of seizures from the age of four months. Diagnostic imaging confirmed the presence of leptomeningeal vascular malformation, calcification in the brain, and abnormal electroencephalogram patterns, establishing the diagnosis of Sturge-Weber syndrome. Treatment with antiepileptic drugs led to seizure control. This case underscores the importance of early diagnosis and tailored treatment strategies for patients with Sturge-Weber syndrome.

## INTRODUCTION

Sturge-Weber syndrome (SWS) is a congenital neurocutaneous syndrome defined by the association of a facial capillary malformation in the trigeminal nerve's ophthalmic distribution with vascular malformation of the brain, eye and ipsilateral vascular glaucoma.^1^ SWS is a rare condition with an incidence of 1 case per 50,000 population.^2^ We present a case of five years old child diagnosed with Sturge-Weber syndrome. The Sturge-Weber syndrome and port-wine stains are thought to be caused by somatic mosaic mutations that interfere with vascular development.^3^

## CASE REPORT

A five-year-old female child, first by birth order and born out of non-consanguineous marriage presented to the paediatrics department with high-grade fever, headache and generalized tonic-clonic type of convulsion for the last 3 days. The patient's mother revealed that the first seizure occurred at 4 months of age and the child was under antiepileptic drugs (AED). The child has not had seizures in the last one year. History also revealed reddish discolouration (port-wine stain) in the face from birth and was gradually darkening with time.

**Figure 1:**
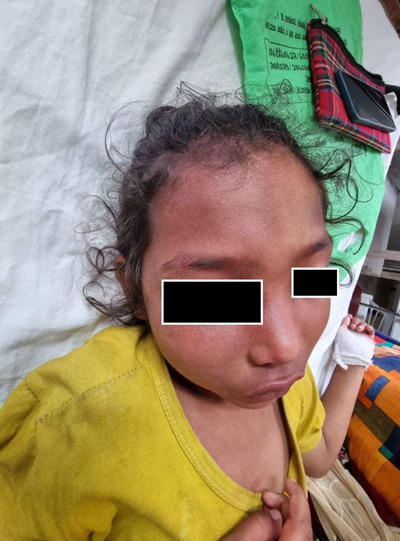
Port-wine stain on the right side of the face (forehead, eyes and nose).

On inspection, it was found that there was a reddish stain on the right side of the forehead that involved the eyes, half of the nose and cheek along the distribution of ophthalmic division of the trigeminal nerve (Figure 1).

A general examination revealed stable vitals and no relevant systemic findings. The child's developmental age lagged behind her chronological age.

Magnetic resonance imaging (MRI) of the brain showed subtle T2 fluid-attenuated inversion recovery (FLAIR) hyperintensity in the right occipitoparietal region. The brain computed tomography (CT) scan showed a reduction in the volume of brain tissue (parenchymal volume loss) and the presence of calcified areas in the white matter on both sides of the brain (bilateral white matter calcification). The electroencephalogram (EEG) performed during sleep showed abnormal patterns, indicating the presence of a possible structural abnormality in the right hemisphere of the brain. The clinical evaluation and imaging modalities confirmed the diagnosis of Sturge-Weber syndrome.

Initially, the child was treated with phenobarbitone and was shifted to sodium valproate at 6 months of age. After the diagnosis of Sturge-Weber syndrome, she was commenced with sodium valproate and oxcarbamazepine (loading and maintenance doses). After three weeks of admission, she became seizure-free. The parents of the child were properly counseled and the child was discharged on oral antibiotics and AED. The patient is regular on follow-up and is remaining seizure-free on AED.

## DISCUSSION

SWS is a rare congenital neurocutaneous disorder with an incidence of 1 case per 50,000 population. Although the aetiology is not well established, a previous study conducted has shown that a somatic activating mutation in the GNAQ gene is responsible for SWS.^3^ The persistence of immature sinusoidal vascular channels and underdeveloped superficial venous drainage with compensatory dilatation of venules leading to shunting of blood to deeper veins, stasis and ischemia ultimately resulting in seizures, transient hemiparesis and progressive deposition of calcium salts is the postulated pathogenesis for SWS.^4^

SWS is characterized by leptomeningeal angiomatosis and port wine stain of the face in the region of the trigeminal nerve's ophthalmic distribution.^4^ Other neurological symptoms include seizures, hemiparesis, recurrent headaches, stroke-like episodes, psychomotor retardation, and mental retardation.^5^ SWS may lead to cognitive impairments, visual field deficits, stroke-like episodes, endocrine problems and learning difficulties. Cutaneous manifestation (port-wine stain) in the distribution of ophthalmic and maxillary divisions of the trigeminal nerve is common in SWS. In 90% of patients with SWS, the first manifestation is seizure (focal or generalized) which occurs in the first year of life. In our case, the first attack of seizure came to the child at the age of four years and the reddish stain was seen in the child from the birth itself which gradually darkened with age.

Radiological imaging with the findings of leptomeningeal vascular malformation on contrast-enhanced T1-weighted magnetic resonance imaging (MRI), and cortical and subcortical calcification on head computed tomography (CT) is the basis of diagnosis.^6^ EEG, magnetic resonance spectroscopy and fluorodeoxyglucose-positron emission tomography (FDG-PET) may also help in the evaluation of patients but are not routinely used and are preferred. MRI is the most preferred modality for diagnosis in patients aged above one. CT scan detects calcification, gyriform calcification being the most common feature and described as a "tram-track sign". The use of ionizing radiation in CT scans limits its use.

The treatment modality is generally supportive and symptomatic management using AED and aspirin is done.^7^ Surgical procedures, which include hemispherectomy, corpus callosotomy, vagal nerve stimulation, focal resection of seizure focus (contraindicated in patients with bilateral involvement), are reserved for people not responsive to medical therapy and patients with glaucoma, refractory seizures and scoliosis. However, most patients achieve seizure control with medications.

This case provides valuable insights into the clinical presentation, diagnosis, and management of SWS in a young patient. The comprehensive assessment, including diagnostic imaging and electroencephalogram, contributed to a well-supported diagnosis. Although this case showcases the successful application of medical management, it doesn't delve into the results of surgical interventions, which, in certain cases resistant to conventional treatment, may be an essential aspect of care.

SWS is a rare sporadic genetic syndrome which lacks a definitive treatment. This case report highlights the importance of recognizing SWS in young patients presenting with seizures and facial port-wine stains. A comprehensive understanding of the latest treatment methods and a thorough knowledge of this syndrome are crucial for effectively managing this condition. The clinical observations in this case enhance our understanding of SWS and emphasize the importance of being proactive in early intervention for such cases.

## References

[1] Dulac O, Lassonde M, Sarnat HB (2013). Handbook of clinical neurology [Internet]..

[2] Tonn JC, Grossman SA, Rutka JT, Westphal M (2006). Neuro-Oncology of CNS tumors [Internet]..

[3] Shirley MD, Tang H, Gallione CJ, Baugher JD, Frelin LP, Cohen B (2013). Sturge-Weber syndrome and port-wine stains caused by somatic mutation in GNAQ.. N Engl J Med..

[4] Bachur CD, Comi AM (2013). Sturge-Weber syndrome.. Curr Treat Options Neurol..

[5] Marana Perez AI, Falco Rojas ML, Puertas Martin V, Dominguez Carral J, Carreras Saez I, Duat Rodríguez A (2017). Analysis of Sturge-Weber syndrome: a retrospective study of multiple associated variables.. Neurol Engl Ed..

[6] Comi AM (2011). Presentation, diagnosis, pathophysiology and treatment of the neurologic features of Sturge-Weber Syndrome.. The neurologist..

[7] Comi AM (2007). Update on Sturge-Weber syndrome: diagnosis, treatment, quantitative measures, and controversies.. Lymphat Res Biol..

